# Nutrition education and behavioral interventions in youth team-sport and related development settings: a systematic review of nutrition knowledge, dietary behaviors, hydration, body composition, and performance

**DOI:** 10.3389/fnut.2026.1939289

**Published:** 2026-08-24

**Authors:** Desirée Victoria-Montesinos, Ana María García-Muñoz, María Teresa Mercader-Ros, Yasmina Jaraba-Salinero, Fernando Figueroa-Morales

**Affiliations:** Faculty of Pharmacy and Nutrition, UCAM Universidad Católica de Murcia, Murcia, Spain

**Keywords:** adolescents, body composition, children, dietary behavior, nutrition education, nutrition knowledge, sport performance, sports nutrition

## Abstract

**Introduction:**

Nutrition knowledge and the ability to translate it into appropriate food and hydration choices are important for health, development, recovery, and performance in young athletes. However, evidence on nutrition interventions in youth team-sport settings remains dispersed across sports, educational formats, and outcome domains. This systematic review aimed to synthesize evidence on nutrition education, hydration education, dietary counseling, digital tools, controlled dietary modification, and multicomponent interventions in child, adolescent, and late-adolescent development settings.

**Methods:**

The review followed PRISMA 2020 guidelines and was registered in PROSPERO (CRD420261441507). PubMed/MEDLINE, Scopus, Web of Science Core Collection, SPORTDiscus/EBSCO, and Cochrane CENTRAL were searched from inception to 3 July 2026. Two reviewers independently screened reports, extracted data, and assessed risk of bias using RoB 2 or ROBINS-I. Because of substantial heterogeneity in interventions and outcomes, findings were synthesized narratively.

**Results:**

Database searches identified 419 records. After duplicate removal and screening, 46 full-text reports were assessed, 19 were excluded, and 27 unique studies were included. Soccer/football predominated, with additional evidence from volleyball, basketball, handball, baseball, hockey, American football, and mixed-sport settings. Nutrition and sports nutrition knowledge showed the most consistent favorable changes. Findings for dietary intake, diet quality, body composition, biochemical markers, and performance were mixed. Hydration interventions showed clearer short-term improvements when education was combined with practical cues, improved fluid access, feedback, or individualized prescriptions.

**Discussion:**

Nutrition interventions in youth team-sport and related development settings may support knowledge acquisition and selected hydration behaviors, but their effects on dietary behavior and physiological outcomes remain uncertain. Confidence in the evidence is limited by small samples, short follow-up periods, heterogeneous measures, reliance on self-report, incomplete fidelity reporting, and risk-of-bias concerns. Future trials should use validated age-appropriate measures, objective outcomes, longer follow-up, prespecified analyses, and implementation strategies involving athletes, families, coaches, schools, and clubs.

**Systematic review registration:**

https://www.crd.york.ac.uk/PROSPERO/view/CRD420261441507, CRD420261441507.

## 1 Introduction

Adequate nutrition during childhood and adolescence is essential for linear growth, pubertal maturation, bone mineral accrual, immune competence, neurocognitive development, and the maintenance of energy availability during training and competition (1–3). In young athletes, these needs are superimposed on structured training, match play, tournaments, travel, school schedules, environmental heat exposure, and recovery demands (1, 2). Team-sport athletes face additional challenges because training load varies by position, competitive calendar, maturation stage, playing time, and tactical demands, while many food decisions remain controlled by parents, schools, clubs, or canteen availability rather than by the athlete alone (1, 4, 5).

The nutritional challenges of young team-sport athletes are not limited to total energy intake. Adequate carbohydrate intake is required to support high-intensity intermittent activity, repeated sprint efforts, technical execution, and recovery between sessions, whereas protein supports growth, tissue repair, and training adaptation (1, 3). Micronutrients such as calcium, vitamin D, and iron are also relevant because adolescence is a critical window for bone mass accrual, blood volume expansion, and growth (1, 2). When energy or carbohydrate intake is chronically insufficient, young athletes may experience fatigue, impaired recovery, poor training adaptation, menstrual dysfunction in females, injury risk, or reduced capacity to sustain school and sport demands (1, 2).

Nutrition knowledge and nutritional health literacy are related but distinct constructs. Nutrition knowledge refers mainly to factual understanding of nutrients, foods, and sport-specific recommendations, whereas nutritional health literacy also encompasses the ability to access, interpret, appraise, and apply nutrition information in real-life contexts (4–6). None of the included studies used a formal multidimensional measure of nutritional health literacy. Accordingly, this review uses nutrition knowledge (or sports nutrition knowledge) for the outcomes actually measured and reserves nutritional health literacy for the broader conceptual framework. This distinction is important in adolescents because knowing what should be eaten does not necessarily mean that the athlete has the autonomy, money, time, cooking skills, family support, or club environment required to translate knowledge into behavior (4–6).

Recent literature indicates that adolescent athletes may have better general than sport-specific nutrition knowledge, with persistent gaps around carbohydrate periodization, hydration, recovery nutrition, supplement use, and the relationship between diet, health, and performance (6). For this reason, nutrition education programs have become a common and relatively low-cost strategy in schools, clubs, academies, and sport camps. However, broader nutrition education literature shows that behavior change depends not only on information provision but also on intervention duration, theoretical grounding, focused objectives, fidelity, self-monitoring, feedback, and environmental support (4, 5).

Hydration is a particularly useful model for the practical application of nutrition knowledge in youth sport. Fluid intake can be linked to visible cues, such as urine color, body-mass change during practice, water-bottle routines, sweat-rate estimation, and access to water during breaks (1, 7, 8). Several included studies evaluated hydration education in youth American football, volleyball, soccer, and mixed athletic settings, reporting favorable changes in knowledge or short-term hydration status when education is combined with objective feedback and practical cues (7, 8). These findings suggest that nutrition education may be more likely to support change when the target behavior is simple, observable, reinforced by coaches, and supported by the sport environment.

Previous reviews have shown that nutrition education interventions in athletes may improve nutrition knowledge, although effects on dietary intake, body composition, and performance are less consistent (9, 10). In team-sport players, Sánchez-Díaz et al. (9) reported promising but heterogeneous effects of nutrition education interventions on eating habits, nutrition knowledge, body composition, and physical performance. More recently, Irwin et al. (10) mapped dietary intake, nutrition knowledge, education interventions, and behavioral influences in youth team-sport athletes, highlighting persistent methodological limitations and the need to advance research and practice in this population. However, previous reviews have either focused on team-sport players irrespective of developmental stage, on nutrition education rather than broader nutritional interventions, or on scoping the field rather than systematically appraising intervention evidence across outcome domains (9, 10). Therefore, a systematic review focused on child, adolescent, and development-phase team-sport athletes and related youth sport contexts, separating knowledge, dietary behavior, hydration, body composition, energy availability, biochemical outcomes, and performance, remains warranted.

The existing intervention literature is heterogeneous. Some studies used posters or web-apps (11, 12), short face-to-face or digital education (13, 14), team-based life-skills programs, practical guidance, or dietary feedback (15–17), controlled dietary modification (18), and multicomponent sport-based education (19). Others evaluated match-week behavior-change strategies or individualized counseling (20, 21), parent-supported goal setting (22), or repeated dietitian-led education (23, 24). Consequently, synthesis requires separating knowledge outcomes from dietary behavior, hydration, body composition, energy availability, biochemical markers, and performance, because these outcomes respond to different levels of intervention intensity and environmental support.

Therefore, the aim of the present systematic review was to synthesize published intervention studies evaluating nutrition education and broader nutritional interventions in child, adolescent, and development-phase athletes participating in team sports or closely related youth sport, school, academy, camp, and sport-development contexts. The review focused on nutrition and sports nutrition knowledge and, where measured, related applied skills; dietary behavior and intake, hydration behavior and status, body composition, biochemical or energy-availability outcomes, and sport performance, while also examining intervention components, intervention intensity, contextual support, and methodological quality.

## 2 Materials and methods

This systematic review was conducted according to PRISMA 2020 guidance (25). The protocol was registered in PROSPERO (CRD420261441507).

The core objectives, databases, intervention scope, outcome domains, and risk-of-bias tools were unchanged from the registered record. During full-text assessment, the registered provisions for predominantly adolescent mixed-age samples were operationalized by retaining mixed U18/U23 development-pathway and mixed-sport samples only as explicitly labeled indirect contextual evidence when they were relevant to youth team-sport nutrition. This was an eligibility clarification rather than a change to the primary review question, and no other protocol deviations occurred.

### 2.1 Information sources and search strategy

PubMed/MEDLINE, Scopus, Web of Science Core Collection, SPORTDiscus via EBSCO, and the Cochrane Central Register of Controlled Trials were searched from database inception to 31 July 2026. No date or language restrictions were applied. Search concepts covered nutritional interventions, children and adolescents, team sports and related youth sport settings, and nutrition-, hydration-, body-composition-, or performance-related outcomes. Boolean operators, truncation, phrase searching, and database-specific field syntax were applied. Complete database-specific search strings, interfaces, dates, and yields are reported in Supplementary Table S1.

### 2.2 Eligibility criteria

Eligibility criteria were defined according to the PICOS framework. No lower chronological age limit was prespecified; the youngest included sample comprised children aged 7 years. Mixed-age samples were eligible when child or adolescent results were separately extractable. Exceptionally, mixed U18/U23 development-pathway samples were retained as indirect contextual evidence when the mean age was within the late-adolescent range and the intervention was directly relevant to youth sport nutrition; these studies were explicitly identified and interpreted separately from direct competitive youth team-sport evidence. Other studies involving adults were eligible only when adolescent results were reported separately. Reports describing the same underlying cohort were linked using authorship, setting, recruitment period, sample size, clusters, intervention content, and ethics information, and were counted as one study to avoid double counting.

Eligible interventions included nutrition or sports nutrition education, hydration education, dietary counseling, individualized dietary recommendations, controlled dietary modification with an educational component, digital or infographic-based education, and multicomponent programs with an identifiable nutrition component. Comparators included no intervention, usual practice, alternative education formats, wait-list controls, or within-participant pre-post comparisons. Eligible outcomes included nutrition or sports nutrition knowledge, applied nutrition-related skills, dietary intake or quality, eating behaviors, hydration knowledge or status, body composition, anthropometry, biochemical or energy-availability markers, and sport performance or physical fitness. Observational studies without an intervention, qualitative-only studies, reviews, conference abstracts, trial registries without results, and supplementation-only trials without education or counseling were excluded.

### 2.3 Study selection

All retrieved references were exported to reference-management software. Duplicates were removed by automated detection followed by manual checking. Two reviewers (DV-M and AMG-M) independently screened titles and abstracts and assessed potentially eligible full texts. Reports with possible sample overlap were compared in detail and linked before determining the number of unique studies. Disagreements were resolved by discussion and consensus; a third reviewer (FF-M) adjudicated when necessary.

### 2.4 Data extraction

Two reviewers (DV-M and AMG-M) independently extracted author and year, country, sport, age and sex, sample size, study design, intervention provider and components, comparator, duration and follow-up, outcome instruments, numerical results when available, statistical approach, and implementation-relevant information. Nutrition-specific components were separated from physical activity, supplementation, or environmental co-interventions when possible. Multiple reports from the same cohort were linked and were not counted as independent studies. Disagreements were resolved by consensus and checked by FF-M when unresolved.

### 2.5 Risk-of-bias assessment

Risk of bias was assessed independently by two reviewers (DV-M and AMG-M). Randomized trials were assessed using the revised Cochrane Risk-of-Bias tool for randomized trials (RoB 2) (26). RoB 2 evaluates five domains: bias arising from the randomization process, deviations from intended interventions, missing outcome data, measurement of the outcome, and selection of the reported result. Overall judgments were calculated according to the RoB 2 algorithm: a trial was judged as low risk only when all domains were low risk; as having some concerns when at least one domain raised some concerns and no domain was high risk; and as high risk when at least one domain was high risk or multiple domains raised concerns that substantially lowered confidence in the result.

Non-randomized controlled studies, quasi-experimental studies, and before-after studies were assessed using ROBINS-I or an adapted ROBINS-I framework according to design (27). Domains considered included confounding, selection of participants, classification of interventions, deviations from intended interventions, missing data, measurement of outcomes, and selection of the reported result. Overall ROBINS-I judgments followed the standard principle that the overall judgment cannot be better than the worst domain-level judgment. Uncontrolled pre-post studies were generally expected to have at least serious concerns for confounding because changes over time could not be separated from maturation, training, seasonality, or other contextual influences.

Domain-level judgments were prepared for visualization using robvis.

### 2.6 Data synthesis

Meta-analysis was not undertaken because sports, participant characteristics, intervention dose, comparators, outcome definitions, instruments, follow-up, and designs were not sufficiently comparable. Before synthesis, studies were tabulated by design, population and context, intervention category, comparator, dose and duration, provider, adherence or fidelity when reported, follow-up, outcome measure, and risk of bias. Findings were then grouped into nutrition and sports nutrition knowledge; dietary behaviors, intake, and diet quality; hydration behavior and status; body composition, anthropometry, biochemical markers, and energy availability; and sport performance or physical fitness. Within each domain, the synthesis compared the direction and magnitude of reported changes when available, exact estimates and significance values, consistency across studies assessing comparable constructs, study design, risk of bias, directness to competitive youth team sport, and objective versus self-reported measurement. Controlled evidence and objective outcomes were given greater interpretive weight, but contradictory findings were retained. The synthesis did not use vote counting based solely on statistical significance, and a non-significant result was not interpreted as evidence of no effect. Nutrition-only interventions were distinguished from multicomponent interventions for which nutrition-specific effects could not be isolated.

Consistent with the registered PROSPERO record, certainty of evidence was not formally rated using GRADE. The review addressed multiple non-equivalent intervention contrasts and broad outcome domains, and most studies did not provide sufficiently comparable numerical effect estimates for a common outcome-specific synthesis. A formal GRADE assessment would therefore have required numerous fragmented ratings without a shared effect estimate or directly comparable body of evidence. Instead, interpretation explicitly considered design, risk of bias, directness, consistency, reported precision, and measurement method.

## 3 Results

### 3.1 Study selection

**Figure 1:**
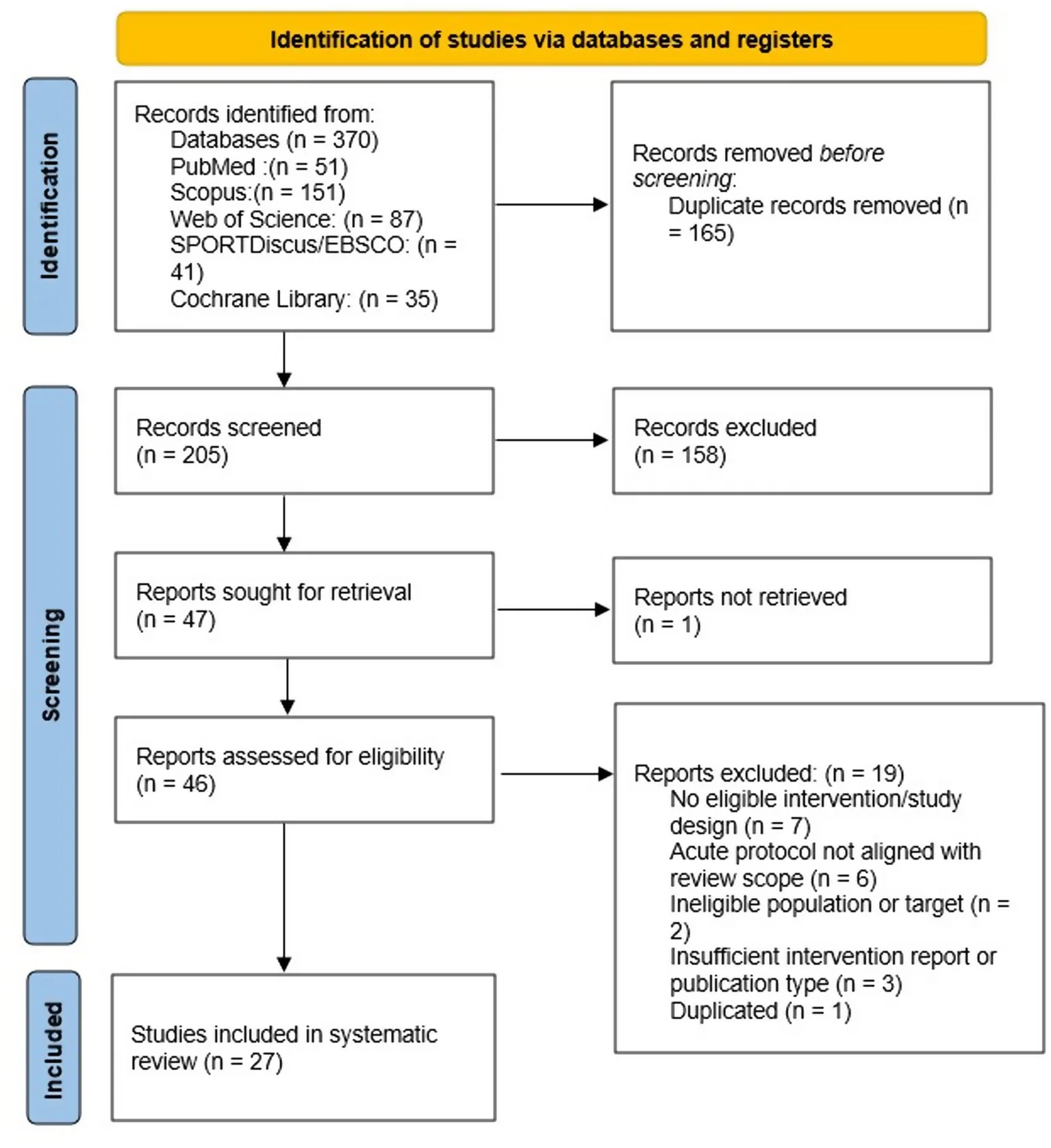
PRISMA 2020 flow diagram of study selection.

The database searches identified 370 records: 56 from PubMed/MEDLINE, 151 from Scopus, 87 from Web of Science, 41 from SPORTDiscus/EBSCO, and 35 from Cochrane CENTRAL. After removal of 165 database duplicates, 205 database records were screened and 158 were excluded at title/abstract stage. In total, 47 reports were sought for retrieval. Forty-six full-text reports were assessed and 19 were excluded: seven for no eligible intervention or design, six for acute protocols outside the review scope, two for ineligible population or target, three for insufficient intervention reporting or publication type, and one as a secondary report of an overlapping cohort. Twenty-seven unique studies (7, 8, 11, 13–24, 28–39) were included. The flow is shown in Figure 1, and excluded and non-retrieved reports are detailed in Supplementary Table S2.

### 3.2 Study characteristics

Across the 27 unique studies, 1,799 athlete participants contributed to final or analyzed samples. One development-phase soccer study included a mixed U23/U18 sample (20); it was retained as indirect development-pathway evidence because U18 outcomes were not reported separately. Restricting the descriptive sample to child/adolescent participants yielded 1,781 athletes. Female participants represented 32.2% of child/adolescent participants with reported sex (537/1,668). Parent participants in the Stewart program were not included in athlete totals. Two Asturias reports described the same five-cluster, 2018–2019 cohort and six-month web-app/poster intervention; Fernández-Álvarez et al. (11) was retained as the primary report, whereas Martín-Payo et al. (12) was classified as an overlapping secondary report and not counted separately.

The studies were published between 1999 and 2026. Soccer/football was most represented (11, 13–15, 17, 19, 20, 29, 32–34, 38), followed by volleyball (31, 35, 36), basketball (39), handball (23, 37), baseball (16), hockey (28), American football (7, 8, 22), and mixed-sport youth settings (18, 21, 24, 30). Mixed-sport and school/recreational studies were treated as contextual rather than direct competitive team-sport evidence.

Intervention duration ranged from a single short educational exposure to 2 years. Longer or repeated programs included the two-year soccer life-skills intervention (15), four-month baseball program (16), six-month Asturias program (11), approximately 8 months of individual counseling in mixed-sport athletes (21), and the six-week WAVE football program with 12-week follow-up (22). Table 1 summarizes populations, intervention components, outcomes, and principal numerical findings. Table 2 provides a concise, non-mutually exclusive grouping of interventions by type and overall pattern of findings.

**Table 1:** Characteristics and main findings of included studies.

Study	Population/context	Design	Intervention	Comparator	Duration/follow-up	Outcomes	Main findings and interpretation
Reading et al. (28)	Canada; adolescent and young adult male hockey players in a sport-camp/education context.	Single-group pre-post intervention	SNAC workbook-based sport nutrition education.	Pre-intervention values/no concurrent comparator.	Short-term camp follow-up.	Sport nutrition knowledge.	Baseline knowledge was low and short-term educational change was limited. The study was retained as early sport-specific educational evidence, although its age range, brief exposure, and limited methodological detail restrict inference.
Leach and Yates (29)	USA; 22 overweight fourth-grade girls in a school/recreational soccer context.	Randomized soccer-versus-wait-list comparison after all participants received the same baseline nutrition education.	A 15-min nutrition-health education session for all participants; intervention arm additionally received recreational soccer.	Wait-list for soccer; not a no-education control.	5 months, including 2 months of soccer exposure.	BMI and nutrition knowledge.	Knowledge increased over time, whereas BMI did not change meaningfully. Because education preceded randomization and was delivered to both groups, the randomized contrast estimates added soccer exposure, not the independent effect of nutrition education.
McDermott et al. (7)	USA; 33 male youth American football campers, approximately 12 years old.	Camp-based controlled intervention	Rehydration education embedded in a football camp using measured hydration and sweat-rate information.	Comparison campers within the same camp setting.	5-day football camp.	Urine osmolality, sweat rate, fluid consumption, hydration practices.	Participants commonly arrived hypohydrated and maintained this status across camp. Education appeared to improve hydration practices, but the camp context limits causal attribution.
Kavouras et al. (30)	Greece; 92 athletic youth, including basketball and volleyball players, training in a warm environment.	Non-randomized controlled intervention	Hydration lecture, urine-color charts, and improved water accessibility in training, dining, and resting areas.	Usual routines/control group.	Pre-post field testing.	First-morning urine specific gravity/osmolality and 600-m run performance.	Hydration markers improved in the intervention group, alongside improved 600-m running performance. The findings suggest that hydration education may be more effective when combined with environmental cues and water availability.
Cleary et al. (31)	USA; 36 elite adolescent female volleyball players in a warm, humid, non-air-conditioned gymnasium.	Within-subject repeated-measures intervention	One slide-based hydration education session followed by an individualized prescribed hydration protocol based on sweat rate.	Within-subject control and observational follow-up periods.	Four observation periods, each including three practices.	Body-mass change, percentage body-mass loss, urine specific gravity, urine color, urine osmolality, sweat rate, fluid volume.	Education alone did not adequately modify hydration behavior. The prescribed hydration protocol was the only condition in which athletes maintained body mass and consumed sufficient fluid to replace sweat losses.
Vilela Silva et al. (36)	Brazil; 10 adolescent female volleyball players from a juvenile team.	Single-group pre-post intervention	Eight monthly meetings addressing nutrition for performance, healthy eating, pressure for results, and self-image.	Pre-intervention values/no concurrent comparator.	Eight monthly meetings.	Nutrition knowledge, intention to change eating behavior, eating attitudes/practices, body dissatisfaction.	Nutrition knowledge increased and six athletes progressed in intention to change eating behavior. Body dissatisfaction did not improve, suggesting that education alone may be insufficient for outcomes involving body image.
Nascimento et al. (21)	Brazil; mixed-sport contextual evidence. Methods and sex totals indicate 21 adolescents aged 12–19 years (6 female) plus 11 adults; adolescent results were reported separately.	Uncontrolled before-after quasi-experimental study.	Four individualized nutrition-counseling consultations at 45–60-day intervals, including education and tailored dietary guidance.	Pre-intervention values; no concurrent control.	8 months.	Nutrition knowledge, dietary behavior/intake, hydration, anthropometry, and body composition.	Adolescent total knowledge increased from 73.6 ± 15.0 to 84.6 ± 11.0 (*p* < 0.05). Body mass, lean mass, and mid-arm muscle area increased (all *p* < 0.05), and meal frequency/water intake improved. Growth, training, selection of completers, and lack of a control limit attribution. The article abstract reverses adult/adolescent group counts; Methods and sex totals support *n* = 21 adolescents.
Patton-Lopez et al. (15)	USA; 217 high-school soccer players; 64% female, 47.5% Latino, mean age 14.9 years; many from low-income settings.	Non-randomized controlled intervention	Team-based sport nutrition education and life-skills curriculum.	Comparison schools by geographic location.	Three assessments across 2 years.	Sports nutrition knowledge, attitudes/beliefs, breakfast/lunch behaviors, eating for performance.	Sports nutrition knowledge increased in the intervention group, and athletes were more likely to report trying to eat for performance. Behavioral changes were selective rather than uniform.
Fernández-Álvarez et al. (11)	Spain; 319 analyzed male soccer players aged 13–16 years from five Asturias teams/clubs.	Cluster-controlled pilot; the primary report describes team randomization, whereas an overlapping secondary report describes non-random club allocation.	Six-month Behavior Change Wheel program using posters, a web-app, practical family activities, coach prompts, goal setting, and rewards.	Control teams/clubs receiving usual practice and information after follow-up.	September 2018 to May 2019.	KIDMED adherence, post-intervention Mediterranean-diet knowledge, feasibility/engagement.	KIDMED did not differ significantly between groups at post-test. Knowledge was higher in IG than CG (3.42 vs. 2.53/5; p < 0.001), but baseline knowledge was not measured, preventing estimation of change. Martín-Payo et al. (12) was treated as an overlapping secondary report and not counted separately.
Zeng et al. (32)	China; 30 elite male youth soccer players, approximately 16–17 years old.	Randomized controlled intervention	Four-week nutrition quality education plus comic book.	Comic book alone.	Four weeks.	KAP questionnaire, food weighing, adjusted Dietary Balance Index.	General nutrition knowledge, sports nutrition knowledge, and total KAP scores improved in the lecture group, whereas attitude, behavior, and DBI indices did not change significantly.
Atkins et al. (8)	USA; 41 in-season high-school American football players, age 16 ± 1 years.	Non-randomized controlled intervention	Five-minute hydration presentation, self-assessment, urine-color charts, water bottles, and hydration-status feedback.	No-intervention team.	Baseline, 3 days, and 24 days post-intervention.	24-h fluid/water consumption, urine specific gravity, hydration knowledge.	Urine specific gravity and water intake improved at 3 days in the intervention group, but effects were attenuated at 24 days, suggesting limited durability after brief education.
Ueda et al. (16)	Japan; 50 male high-school baseball players from two public high schools.	Non-randomized controlled intervention	CAN-framework nutrition education with lectures, individual guidance, food-photo recording, dietary behavior plans, goal setting, and bento-box activities.	Control school without program.	Four-month intervention.	Food and beverage intake, dietary balance, body mass/lean mass, bat swing speed, qualitative experiences.	The intervention improved food intake, dietary balance, and bat swing speed. The program combined education with repeated practical and individualized components.
Sánchez-Díaz et al. (9)	Spain; 23 elite U14 basketball players from a professional academy.	Single-group pre-post intervention	Five nutrition education sessions combined with a weekly FIFA 11 + program.	Pre-intervention values/no concurrent comparator.	Five months.	Fitness tests, PAQ-A, eating/nutritional habits, nutrition knowledge.	Physical fitness and physical activity improved, while eating habits and nutrition knowledge were maintained. Attribution to nutrition education is limited because the intervention also included FIFA 11 + .
Gao et al. (33)	China; 41 U15 male soccer players.	Non-randomized comparative intervention	Classroom-based nutrition education.	WeChat public-account article learning with the same content.	12 weeks, with immediate, 6-week, and 12-week follow-up.	Athlete nutrition KAP questionnaire and self-efficacy questionnaire.	Both formats improved self-efficacy and knowledge, but classroom teaching produced greater and more sustained gains than WeChat article learning.
Grabia et al. (17)	Poland; 46 young soccer academy players aged 12–17 years.	Non-randomized comparative intervention	Individual recommendations plus additional group nutrition education.	Individual recommendations only.	17 weeks.	Nutrient intake, eating habits, fluid intake, biochemical parameters, wellbeing and perceived recovery.	Additional group education improved selected dietary habits, water intake, peri-workout hydration routines, wellbeing, and perceived recovery compared with individual recommendations alone.
Morgado et al. (34)	Portugal; 67 schoolchildren aged 7–10 years.	Non-randomized controlled intervention	Recreational football plus nutrition education.	Recreational football only and usual curriculum control.	12 weeks.	Body composition, physical fitness, physical activity, blood pressure, eating behaviors, nutrition knowledge, psychosocial health.	The nutrition-plus-football group improved nutritional knowledge, fruit and fish consumption, waist-to-height ratio, blood pressure, and several fitness/health markers. Interpretation should consider the combined football and nutrition components.
Stewart et al. (22)	USA; 25 high-school American-football athletes (mean age 15.92 ± 1.26 years; 24 male, 1 female) and one parent per athlete.	Single-group quasi-experimental pilot with convenience sampling.	Six weekly WAVE sport-nutrition lessons adapted for football, delivered by graduate dietetics students, with athlete goal setting and optional parent participation.	Pre-intervention values; exploratory comparison by parent attendance; no concurrent control.	6 weeks plus 12-week follow-up.	Sports nutrition knowledge, dietary screeners, fat-free mass, goal attainment; parent knowledge and self-efficacy.	Among completers, knowledge increased at 6 weeks (13.22 ± 4.41 to 21.56 ± 9.29; *p* < 0.01) but not 12 weeks (*p* = 0.38). Carbohydrate score improved at 6 weeks (*p* = 0.01) but not 12 weeks; diet quality and fat-free mass did not change. Only 64% of athlete/parent participants completed 12-week follow-up, and parent attendance was not associated with athlete goal adherence.
Debnath et al. (18)	India; 105 male athletes aged 14–18 years from football, hockey, swimming, and athletics.	Randomized controlled intervention	Eight-week nutrition education program plus controlled dietary modification.	Ad-libitum diet/no structured nutritional intervention.	8 weeks.	Nutrition knowledge, nutrition practice, daily food intake, anthropometry.	Knowledge, nutrition practice, and macro- and micronutrient intake improved in the intervention group. The study was interpreted cautiously because the sample included both team and individual sports and the intervention combined education with controlled dietary modification.
Kurdanti et al. (38)	Indonesia; adolescent male soccer athletes from athlete dormitory/training settings.	Non-randomized controlled intervention	Nutrition education, counseling, meal-portion assistance, and vitamin D supplementation.	Comparison/control group.	1-month intervention; study conducted April–August 2019.	Nutrient intake, body composition, vitamin D status, creatine kinase, VO₂max.	Vitamin D status and selected nutritional/body-composition indicators improved. Attribution to education alone is limited because the intervention also included supplementation and dietary assistance.
Tektunalı Akman et al. (24)	Turkey; 83 female athletes aged 15–18 years from football, basketball, and volleyball, training >10 h/week.	Randomized controlled intervention	Six face-to-face nutrition education lectures delivered by a registered dietitian.	No nutrition education.	6-month follow-up.	Energy availability, LEAF-Q, EAT-26, sports nutrition knowledge questionnaire, 3-day food records, body composition.	Sports nutrition knowledge and energy availability increased, LEAF-Q scores decreased, and energy intake, body weight, fat-free mass, and resting metabolic rate improved in the intervention group. Eating attitude scores did not change significantly.
Carter et al. (20)	England; late-adolescent/development-phase male soccer players; mean age 18.4 ± 1.0 years.	Randomized controlled intervention	Targeted nutrition education and behavior-change strategies focused on match-week fueling, including education, prompts, infographics, nutritionist presence, and recovery support.	No nutrition support.	Match-week intervention/assessment period.	Daily dietary intake across match week and resting metabolic rate.	Intervention players improved energy and carbohydrate intake and match-week dietary practices. The study was retained as late-adolescent/development-phase evidence.
Aytekin and Koç (35)	Turkey; 40 volleyball players aged 9–14 years living in a socioeconomically disadvantaged area.	Single-group pre-post intervention	Three-week nutrition education delivered in three modules.	Pre-intervention values/no concurrent comparator.	3 weeks.	Nutrition habits questionnaire, 24-h food record, Diet Quality Index-International.	Nutrition knowledge improved significantly, whereas body weight and diet-quality scores did not change significantly. The authors related limited dietary change partly to socioeconomic constraints and family food availability.
Şentürk et al. (13)	Turkey; 25 male adolescent football players aged 15–17 years.	Single-group pre-post intervention	Six-week face-to-face sports nutrition education module.	Pre-intervention values/no concurrent comparator.	6 weeks.	Eating habits, eating awareness, adolescent eating behavior perception, athlete nutrition knowledge.	Sports nutrition knowledge improved significantly and post-training eating times improved, but broader eating behavior effects were less pronounced.
Vicente et al. (14)	Portugal; 38 male youth football players aged 13–18 years training at least three times per week.	Single-group pre-post intervention	Three face-to-face educational sessions plus two digital infographics.	Pre-intervention values/no concurrent comparator.	Short-term post-intervention reassessment.	KIDMED, general nutrition knowledge, sports nutrition knowledge, anthropometry/body composition.	General and sports nutrition knowledge improved, but KIDMED scores did not change significantly. The findings suggest knowledge gains without clear short-term change in Mediterranean diet adherence.
Veloso-Pulgar and Farran-Codina (23)	Spain; 45 female handball players, age 17.6 ± 2.1 years.	Single-group pre-post intervention	Three-week program with six in-person sessions delivered by a registered dietitian.	Pre-intervention values/no concurrent comparator.	Immediate and 3-month follow-up.	Sports nutrition knowledge, dietary intake, Mediterranean diet adherence, body composition.	Knowledge improved immediately and at 3 months. Sugary-food consumption decreased, but energy/carbohydrate intake, Mediterranean diet adherence, and body composition changed little. The pilot informed a later cluster RCT.
Simsek et al. (19)	Turkey; 59 adolescent male soccer players aged 10–15 years.	Single-group pre-post intervention	Six-week sports-based nutrition education on adequate, balanced, and sustainable nutrition.	Pre-intervention values/no concurrent comparator.	6 weeks.	Eating Habits Questionnaire for Adolescents; height, weight, waist/hip/thigh circumference, BMI.	Eating habit scores improved significantly. Anthropometric changes were statistically significant but difficult to interpret because growth and maturation were uncontrolled.
Veloso-Pulgar and Farran-Codina (37)	Spain; 97 adolescent female handball players aged 12–19 years from four clubs.	Cluster-randomized controlled intervention	Three 30-min face-to-face group sessions delivered by a registered dietitian.	Control clubs with no nutrition education during the study period.	Immediate post-intervention and 3-month follow-up.	Sports nutrition knowledge, dietary intake, Mediterranean diet adherence, anthropometry/body composition.	Sports nutrition knowledge improved substantially and remained higher at follow-up. Dietary intake, Mediterranean diet adherence, and body composition did not change significantly, suggesting that brief education improved knowledge but was insufficient to modify dietary or body-composition outcomes.

BMI, body mass index; CAN, Convenient, Attractive, Normative framework; CG, control group; CK, creatine kinase; DBI, Dietary Balance Index; EAT-26, Eating Attitudes Test-26; FIFA 11+, injury-prevention warm-up program; IG, intervention group; KAP, knowledge, attitudes, and practices; KIDMED, Mediterranean Diet Quality Index for children and adolescents; LEAF-Q, Low Energy Availability in Females Questionnaire; MD, Mediterranean diet; PAQ-A, Physical Activity Questionnaire for Adolescents; RCT, randomized controlled trial; RMR, resting metabolic rate; SNAC, Sports Nutrition for Athletes and Coaches; SNK, sports nutrition knowledge; USG, urine specific gravity; VO₂max, maximal oxygen uptake.

**Table 2:** Summary of intervention categories and patterns of findings.

Intervention category	Defining components	Representative studies	Overall pattern in this review
Brief or group education	Short lectures, classroom sessions, workbooks, posters, web-apps, or digital infographics.	(11, 13, 14, 23, 28, 32, 33, 35–37)	Nutrition knowledge showed the most consistent favorable changes; dietary intake, diet quality, and body composition changed less consistently.
Individualized counseling or feedback	One-to-one guidance, food-record or photographic feedback, practical tasks, and goal setting, usually delivered repeatedly.	(15–17, 20, 21, 24)	More favorable dietary signals were reported in some studies, but repeated contact and non-randomized designs often limited causal attribution.
Hydration-focused	Education combined with urine-color charts, improved fluid access, water bottles, hydration feedback, or sweat-rate-based prescriptions.	(7, 8, 11, 30, 31)	The clearest short-term objective changes occurred when practical cues, access, feedback, or individualized prescriptions accompanied education; durability remained uncertain.
Digital or infographic-based	Web-apps, WeChat articles, digital infographics, or digital reinforcement added to face-to-face education.	(11, 14, 33)	Digital delivery supported knowledge acquisition, but interactive classroom delivery could produce stronger or more sustained gains than digital-only formats.
Multicomponent, environmental, or parent-supported	Education combined with controlled dietary modification, sport/fitness programs, supplementation, environmental change, or family/parent involvement.	(18, 19, 22, 29, 34, 38, 39)	Broader outcomes sometimes changed favorably, but the active nutrition-education component could not usually be isolated.

### 3.3 Effects of interventions by outcome domain

#### 3.3.1 Nutrition and sports nutrition knowledge

Nutrition or sports nutrition knowledge was the outcome with the most consistent favorable changes. No included study used a formal multidimensional measure of nutritional health literacy. Improvements were reported after workbook, interdisciplinary, team-based, lecture, classroom, digital, repeated-counseling, and dietitian-led programs (11, 13–15, 18, 21–24, 32–37). In the IDEHA-F report, post-intervention knowledge was higher in the intervention than control group (3.42 vs. 2.53/5; *p* < 0.001), but baseline knowledge was not measured, so change attributable to the intervention could not be established (11). Nascimento et al. reported an increase in total knowledge among the adolescent subgroup (73.6 ± 15.0 to 84.6 ± 11.0; *p* < 0.05) (21). In Stewart et al., sports nutrition knowledge increased at 6 weeks among nine completers (13.22 ± 4.41 to 21.56 ± 9.29; *p* < 0.01), but not at 12 weeks (14.50 ± 5.18 to 15.42 ± 3.95; *p* = 0.38) (22). These small complete-case analyses limit certainty about durability.

The magnitude and durability of knowledge gains appeared to depend on intervention intensity and mode of delivery. Gao et al. showed that both WeChat and classroom education improved outcomes, but classroom teaching produced stronger and more sustained knowledge gains (33). Veloso-Pulgar and Farran-Codina found that brief dietitian-led education improved sports nutrition knowledge immediately and at follow-up in female handball players, both in the pilot study and in the subsequent randomized controlled trial (23, 37). However, knowledge improvement alone did not guarantee improvements in diet quality, dietary intake, or body composition (11, 14, 23, 32, 35, 37).

#### 3.3.2 Dietary behaviors, dietary intake, and diet quality

Effects on dietary behaviors and diet quality were more heterogeneous. Repeated or practical interventions improved selected meal patterns, food groups, water intake, peri-training practices, or eating-for-performance behaviors (13, 15–23, 34). Nascimento et al. reported improved meal frequency and daily water intake in adolescents after individualized counseling, alongside favorable changes in fruit, vegetable, sweet, and oil intake, although there was no concurrent control (21). Stewart et al. found a short-term increase in carbohydrate-intake score at 6 weeks (3.33 ± 2.23 to 4.80 ± 2.48; *p* = 0.01), but no difference at 12 weeks (2.73 ± 1.87 to 2.33 ± 1.76; *p* = 0.52), and diet-quality scores did not change (22). These findings reinforce the distinction between short-term knowledge acquisition and sustained dietary change.

In contrast, multiple interventions improved knowledge without significant improvements in diet quality or intake. Zeng et al. (32) improved knowledge but did not significantly change dietary behavior or adjusted Dietary Balance Index scores. Aytekin and Koç (35) reported increased nutrition knowledge in adolescent volleyball players living in a disadvantaged area, but no significant change in Diet Quality Index scores. Vicente et al. (14) improved nutrition knowledge in young football players, but Mediterranean diet adherence did not significantly change. Similarly, Veloso-Pulgar and Farran-Codina (23, 37) improved sports nutrition knowledge in female handball players, but dietary intake, Mediterranean diet adherence, and body composition did not change significantly.

Studies with clearer dietary signals generally included repeated contact, controlled dietary modification, individual counseling, food-record or photographic feedback, goal setting, or practical tasks (16–22). These components may support behavior change but also complicate attribution. Debnath et al. combined education with controlled dietary modification (18), while Kurdanti et al. combined education with vitamin D supplementation (38); physiological changes therefore cannot be attributed solely to education.

#### 3.3.3 Hydration behavior and hydration status

Hydration-focused interventions provided the clearest short-term behavioral and physiological signals. McDermott et al. (7) found that youth football campers arrived hypohydrated and tended to remain hypohydrated across camp, although education appeared to improve hydration practices. Kavouras et al. (30) combined a hydration lecture with urine-color charts and increased water accessibility, leading to improved urine specific gravity and osmolality and improved 600-m run performance in the intervention group. Atkins et al. (8) used a brief education session, urine-color charts, water bottles, and hydration feedback in high-school football players, improving urine specific gravity and water intake at 3 days, although effects were not maintained at 24 days.

Cleary et al. provided an important contrast between education and individualized prescription in elite adolescent volleyball players (31). A one-time educational session alone was insufficient to maintain body mass during practice, whereas an individualized sweat-rate-based hydration prescription was associated with better maintenance of body mass. Across hydration studies, practical access, feedback, and prescribed routines appeared more influential than information alone.

#### 3.3.4 Body composition, anthropometry, biochemical markers, and energy availability

Body-composition and anthropometric outcomes were inconsistent and require cautious interpretation. Several studies reported favorable changes in weight-related measures, fat-free mass, or circumferences (19, 21, 24, 34, 38). In the adolescent subgroup of Nascimento et al., body mass (56.1 ± 10.2 to 58.0 ± 8.7 kg), lean mass (46.9 ± 8.4 to 48.1 ± 7.5 kg), and mid-arm muscle area (45 ± 12 to 51 ± 15 cm^2^) increased (all *p* < 0.05), but growth, training, and lack of a comparator preclude attribution to counseling (21). Stewart et al. reported no significant change in fat-free mass at 12 weeks (*p* = 0.41) (22). Multicomponent studies likewise could not isolate nutrition-specific effects.

However, body composition results in youth sport interventions should not be overinterpreted. Children and adolescents are growing, and changes in weight, BMI, fat mass, lean mass, and circumference can reflect maturation, training adaptation, seasonality, or measurement error rather than nutrition education effects. This is especially relevant in single-group pre-post studies (13, 19, 23, 35, 39). Biochemical outcomes were also heterogeneous. Grabia et al. (17) assessed biochemical parameters in young soccer players but the most interpretable changes related to dietary habits and selected nutrient intakes. Kurdanti et al. (38) included vitamin D supplementation, which confounds attribution of improvements in vitamin D status and related body-composition outcomes to nutrition education.

#### 3.3.5 Sport performance and physical fitness

Sport performance and physical fitness outcomes were assessed less frequently than knowledge or dietary outcomes. Kavouras et al. (30) reported improved 600-m running performance after improved hydration status. Ueda et al. (16) reported increased bat swing speed after the CAN-framework nutrition education program in baseball players. Sanchez-Diaz et al. (39) reported improvements in countermovement jump, agility, and repeated-change-of-direction performance after nutrition education combined with the FIFA 11 + program in young basketball players. Morgado et al. (34) observed improvements in physical fitness outcomes after football-based programs.

### 3.4 Risk of bias and reporting considerations

RoB 2 assessments of the randomized studies yielded overall judgments of some concerns, whereas ROBINS-I assessments of non-randomized and pre-post studies ranged from moderate to serious. The existing domain judgments for the originally assessed studies were retained unchanged. Nascimento et al. and Stewart et al. were judged at serious overall risk: Nascimento et al. because of uncontrolled before-after comparisons, selection of completers, and self-reported outcomes (21); and Stewart et al. because of convenience sampling, absence of a concurrent control, substantial complete-case attrition, and self-reported outcomes (22).

Moderate judgments in the original ROBINS-I set generally occurred when studies included comparator groups, structured delivery, repeated assessments, objective hydration or anthropometric measures, or more detailed dietary assessment, although residual confounding remained (7, 8, 11, 16, 17, 30, 34, 38).

Serious judgments were principally driven by single-group pre-post designs, fixed intervention sequences, non-random allocation, missing data, self-reported outcomes, and inability to distinguish nutrition effects from maturation, training, seasonality, test familiarization, or co-interventions (13–15, 19, 21–23, 28, 31, 33, 35, 36, 39).

Overall, randomized evidence raised some concerns, while non-randomized evidence commonly had moderate or serious risk of bias. Domain-level judgments are shown in Supplementary Figures S1–S3.

## 4 Discussion

This systematic review synthesized 27 unique intervention studies evaluating nutrition education, hydration education, dietary counseling, digital tools, controlled dietary modification, and multicomponent programs in youth team-sport and related development settings. Nutrition and sports nutrition knowledge showed the most consistent favorable changes, whereas dietary intake, diet quality, hydration status, body composition, energy availability, biochemical markers, and performance varied according to intervention intensity, behavioral support, context, and measurement. This pattern is consistent with previous reviews (9, 40–42), but the present synthesis further distinguishes direct competitive team-sport evidence from mixed-sport, school, and development-context evidence.

The clearest pattern was the gap between knowledge and sustained behavior. Most studies measuring knowledge reported improvement (11, 13–15, 18, 21–24, 32–37), but dietary behaviors changed less consistently. This pattern was also evident in Nascimento et al., who reported favorable before-after changes without a comparator (21), whereas Stewart et al. observed six-week gains that were not maintained at 12 weeks and had substantial attrition (22). Knowledge should therefore be interpreted as an intermediate outcome rather than evidence of durable dietary change.

This interpretation is also supported by adult and collegiate athlete studies. For example, Valliant et al. (43) showed that individualized support from a registered dietitian improved both nutrition knowledge and dietary intake in NCAA female volleyball players, whereas Alaunyte et al. (44) found that professional rugby league players with higher nutrition knowledge consumed more fruit, vegetables, and carbohydrate-rich foods, but that knowledge did not translate uniformly across all dietary domains. These findings mirror the present review: when education is linked to individual feedback, counseling, or practical dietary targets, dietary change is more likely; when knowledge is assessed in isolation, the relationship with actual intake is weaker. Therefore, the adolescent team-sport literature appears to follow the same pattern observed in older athletes, but with additional developmental and environmental barriers.

Intervention intensity may be one factor influencing behavioral transfer. Brief lectures, posters, workbooks, web-apps, articles, and infographics were feasible and were often accompanied by knowledge gains (11–14, 28, 33), but were less often accompanied by robust dietary change when used alone. This pattern is consistent with Boidin et al. (41), who found inconsistent dietary changes across heterogeneous athlete nutrition-education programs. That review also highlighted poor-to-fair study quality and frequent use of self-reported dietary assessment, limitations also observed here.

Interventions incorporating individual counseling, repeated sessions, food-record review, photographic feedback, goal setting, practical food tasks, or controlled dietary modification showed more favorable dietary signals (15–18, 20, 24), although these components and the predominance of non-randomized designs limited attribution. The findings suggest that future programs may benefit from moving beyond one-directional information transfer and incorporating self-monitoring, feedback, environmental restructuring, action planning, and stakeholder support. In youth sport, parents, coaches, team managers, and school food providers are part of the intervention pathway because they influence food access and opportunities for behavior change.

Implementation also varied markedly between studies. Providers ranged from teachers, coaches, and graduate trainees to registered dietitians; planned exposure ranged from a single brief session to repeated individual consultations; and parental or coaching involvement and environmental support were inconsistent (7, 8, 11, 15–18, 20, 22–24, 30, 31, 33). Few studies reported the planned versus delivered dose, attendance, participant engagement, adherence, protocol adaptations, or fidelity checks in sufficient detail. Accordingly, a null finding may reflect limited reach or implementation as well as an ineffective intervention concept, while a favorable multicomponent finding cannot identify which component was active. These implementation differences likely contributed to between-study heterogeneity and limit direct comparisons between programs.

Hydration interventions illustrate this principle particularly well. Hydration behaviors are easier to operationalize than overall diet quality because they can be linked to immediate and visible feedback: urine color, body-mass loss, water-bottle use, sweat-rate estimation, and fluid availability. Kavouras et al. (30) improved hydration markers and running performance by combining education with urine-color charts and improved water access. Atkins et al. (8) showed a short-term improvement after education, feedback, charts, and water bottles, but the effect weakened by 24 days. Cleary et al. (31) showed that individualized sweat-rate-based prescriptions were more effective than education alone in maintaining body mass during volleyball practice. These findings are consistent with the team-sport hydration review by Nuccio et al. (45), which concluded that hypohydration may impair cognitive, technical, and physical performance, particularly at higher levels of body-mass loss and under heat stress. However, the present review adds an implementation-relevant point: hydration education showed clearer signals when the target behavior is converted into a routine embedded in practice structure and reinforced by coaches, rather than delivered as isolated information.

The weaker and more heterogeneous effects on diet quality should also be interpreted in context. Mediterranean diet adherence, Dietary Balance Index scores, Diet Quality Index scores, and food-group consumption are broad outcomes influenced by cultural norms, household food purchasing, affordability, cooking skills, school cafeterias, and peer environments. Aytekin and Koç (35) showed improved knowledge without diet-quality improvement in volleyball players living in a disadvantaged area, highlighting that education cannot fully overcome structural constraints. Vicente et al. and Veloso-Pulgar and Farran-Codina similarly improved knowledge without significant change in Mediterranean diet adherence (14, 23, 37). These findings are consistent with broader literature on athletes’ food choice, which indicates that taste, convenience, cost, time availability, sport culture, and body-composition pressures influence intake alongside knowledge (46). They are also aligned with Spronk et al. (47), who showed that the relationship between nutrition knowledge and dietary intake is positive but inconsistent across populations. Therefore, when the outcome is broad diet quality, athlete-only education may be insufficient unless it is accompanied by family engagement, food-environment change, and practical strategies adapted to the athlete’s resources.

Female athlete studies require particular attention because knowledge gains may not be enough to reduce low energy availability or body-image-related risk. Tektunalı Akman et al. (24) reported improvements in energy availability and LEAF-Q scores after dietitian-led education, whereas the handball studies by Veloso-Pulgar and Farran-Codina (23, 37) improved sports nutrition knowledge but did not significantly change energy intake, carbohydrate intake, Mediterranean diet adherence, or body composition. These findings are complementary rather than conflicting. They suggest that female athlete interventions may need more than brief education, particularly when the target is energy availability, menstrual health, or carbohydrate adequacy. This interpretation is consistent with the 2023 IOC consensus statement on REDs, which emphasizes the multifactorial nature of low energy availability, its potential adverse effects on health and performance, and the need for prevention, screening, education, and multidisciplinary management (48) as well as with a systematic review of educational interventions reporting favorable but heterogeneous effects on nutrition knowledge and fueling behaviors (49). Therefore, programs targeting female adolescent athletes should include repeated individualized counseling, monitoring of menstrual and training symptoms, and involvement of qualified dietitians when the objective is to change intake rather than only improve knowledge (1, 23, 24, 37, 48, 49).

Body composition and performance outcomes were the least straightforward endpoints. Some studies reported favorable changes in anthropometry or fitness (19, 24, 34, 38, 39), but most did not isolate nutrition education from growth, maturation, training effects, supplementation, or concurrent exercise programs. For example, Sanchez-Diaz et al. (39) combined nutrition education with FIFA 11+, Morgado et al. (34) combined football with nutrition education, Kurdanti et al. (38) combined education with vitamin D supplementation, and Debnath et al. (18) combined education with controlled dietary modification. This partly explains why the team-sport review by Sánchez-Díaz et al. found promising effects on knowledge, eating habits, and body composition, but more disparate effects on performance (9). Similar inconsistency has been reported in broader athlete reviews, where dietary intake may improve without clear or immediate effects on body composition or performance (41, 49). These designs are valuable from a pragmatic perspective because real-world sport programs are often multicomponent. However, they limit causal attribution. Future trials should measure maturation status, training load, baseline nutritional status, pubertal stage, and objective dietary or hydration endpoints so that changes in body composition or performance can be interpreted mechanistically rather than attributed automatically to education.

Digital education is promising but should not be treated as equivalent to interactive education. Gao et al. (33) directly compared WeChat article-based learning with classroom education and found stronger and more consistent effects with classroom teaching. Vicente et al. (14) used face-to-face sessions plus digital infographics and improved nutrition knowledge. These findings are consistent with Heikkilä et al. (50), who showed that young endurance athletes improved nutrition knowledge after education and food-diary feedback, but that adding a mobile application did not provide additional learning benefit. Digital tools may therefore be useful for reinforcement, scalability, reminders, and family communication, but they may need to be combined with interactive discussion, practice-based examples, feedback, and coach support to change behavior. This is particularly relevant in adolescents, whose attention, autonomy, and food choices are shaped by digital information but also by immediate social environments.

The methodological quality of the evidence base limits certainty. Several studies were small pilots (14, 22, 23, 29), and many non-randomized or pre-post studies lacked a concurrent control (13, 14, 19, 21–23, 28, 31, 35, 36, 39). Even randomized trials incompletely reported allocation concealment, blinding, fidelity, attrition, or prespecified primary outcomes (18, 20, 24, 29, 32, 37). Follow-up was often short, further limiting inference about durability.

Differences in dietary assessment also contributed to heterogeneous findings. Included studies used combinations of 24-h recalls, one- or multi-day food records, food-frequency or screening questionnaires, photographic records, and diet-quality indices (14–18, 21–24, 32, 35, 37). These methods differ in the days captured, coverage of training and rest days, portion-size estimation, and sensitivity to short-term change. Self-report is also vulnerable to reactivity, recall and social-desirability bias, and under-reporting; Capling et al. (51) found that self-reported energy intake in athletes is frequently underestimated and that validation evidence is limited. Consequently, null or inconsistent dietary findings may partly reflect measurement error rather than absence of behavioral change.

These limitations are not unique to the present review. Tam et al. and Trakman et al. (40, 52) highlighted substantial heterogeneity and limited validation of athlete nutrition-knowledge instruments, while Hulland et al. (6) found that adolescent-athlete studies often used inadequately validated questionnaires and lacked sample-size justification. The field therefore supports stronger descriptive conclusions about knowledge acquisition than about sustained dietary change, body composition, biochemical markers, or performance.

From an implementation perspective, the review supports a stepped model. First, programs should assess the athlete’s baseline knowledge, training schedule, food environment, hydration routines, maturation stage, sex-specific risk factors, and available support. Second, education should focus on a small number of sport-specific behaviors that are actionable for the athlete’s age and context. Third, programs should add behavioral supports: meal examples, shopping or snack lists, practice-day routines, sweat-rate or urine-color feedback, recovery checklists, and individual feedback from food records or photographs. Fourth, parents and coaches should be involved because they control or influence many of the opportunities for behavior change. Finally, outcomes should be measured after a sufficient period to determine whether knowledge has translated into sustained routine, and not only immediately after the education session.

Overall, the evidence suggests that nutrition education is necessary but not sufficient. It is necessary because many young athletes have gaps in sport-specific nutrition knowledge (6, 15, 28, 32, 33). It is not sufficient because diet and hydration behaviors are embedded in social, economic, developmental, and sport-specific environments (1, 4, 5, 46, 47). Compared with previous systematic reviews in broader athletic populations (9, 40–42), the present review suggests that these challenges may be amplified in adolescent team-sport contexts, where food choices are rarely controlled by the athlete alone. The interventions showing the clearest favorable signals tended to combine accurate education with practical skill development, environmental support, individualized feedback, and repeated reinforcement across the athlete’s home, school, and club settings.

### 4.1 Strengths and limitations of the review

A strength of this review is that it integrates nutrition education, hydration education, dietary counseling, digital tools, and multicomponent programs across several team sports and youth contexts. The review separates knowledge outcomes from behavioral, physiological, and performance outcomes, which is essential for interpreting the intervention pathway. It also explicitly evaluates risk of bias using tools matched to study design and distinguishes nutrition-only interventions from multicomponent interventions.

Compared with Sánchez-Díaz et al. (9), who reviewed nutrition education interventions in team-sport players, and Irwin et al. (10), who mapped dietary intake, nutrition knowledge, education interventions, and behavioral influences in youth team-sport athletes, the present review provides a broader intervention-focused synthesis. Its added value lies in including hydration education, dietary counseling, controlled dietary modification, and multicomponent programs, and in separating knowledge outcomes from dietary, hydration, body-composition, energy-availability, biochemical, and performance outcomes.

The review is limited by heterogeneity, small samples, incomplete intervention reporting, predominance of pilot and non-randomized designs, and inability to perform meta-analysis. Some studies represented mixed-sport, recreational, school, or older development-phase contexts and were therefore indirect for competitive youth team sport. The electronic strategy used named sports and an outcome block, which may have reduced sensitivity; therefore, some risk of missed eligible reports remains. Several interventions combined education with training, supplementation, dietary modification, or environmental support, limiting causal attribution. One report could not be retrieved. Formal GRADE ratings were not generated; therefore, the review does not assign certainty categories and supports descriptive conclusions rather than graded recommendations. These limitations require cautious interpretation of positive findings.

## 5 Conclusion

Nutritional interventions in youth team-sport and related sport-development contexts may improve nutrition and sports nutrition knowledge and, in some contexts, may also improve hydration behavior, dietary practices, energy availability, or selected performance-related outcomes. However, knowledge gains do not reliably translate into dietary behavior change unless education is repeated, practical, sport-specific, and supported by feedback, families, coaches, schools, or clubs.

Hydration education provided the clearest applied model because practical cues and objective markers can be linked to immediate behavior. Broader dietary outcomes may require more intensive and environmentally supported interventions. Overall certainty remains limited by small samples, short follow-up, heterogeneous outcomes, and RoB 2 judgments of some concerns or ROBINS-I judgments of moderate or serious risk. Future research should use rigorous theory-informed designs, longer follow-up, validated age-appropriate nutrition-knowledge measures and, where relevant, measures of applied skills, objective outcomes, prespecified analyses, and implementation strategies reflecting young athletes’ food and sport environments.

## Data Availability

The original contributions presented in the study are included in the article/Supplementary material, further inquiries can be directed to the corresponding author.

## Supplementary material

Supplementary Figure S1 RoB 2 judgments for individually randomized trials.

Supplementary Figure S2 RoB 2 judgments for the cluster-randomized trial.

Supplementary Figure S3 ROBINS-I judgments for non-randomized and pre-post studies.
