# Supplementary material for: Nutrition education and behavioral interventions in youth team-sport and related development settings: a systematic review of nutrition knowledge, dietary behaviors, hydration, body composition, and performance
Source: Front Nutr. 2026 Aug 24;13:1939289. doi: 10.3389/fnut.2026.1939289 (PMC13547255; doi:10.3389/fnut.2026.1939289)
Supplement: Supplementary file 1 [file Table_1.docx]

**Table S1.** Complete database-specific search strategies and study-selection yield.

Search date: 31 July 2026. Coverage: database inception to the search date. No date or language restrictions were applied. Search strings are reproduced below with the quotation marks, truncation, Boolean operators, and field codes used in each interface.

Complete database-specific syntax and exported records

| Database/source | Date searched | Complete search syntax | Records identified/exported (n) |
| --- | --- | --- | --- |
| PubMed/MEDLINE | 31 July 2026 | ("nutrition education"[Title/Abstract] OR "nutritional education"[Title/Abstract] OR "sports nutrition education"[Title/Abstract] OR "dietary intervention"[Title/Abstract] OR "nutrition intervention"[Title/Abstract] OR "nutrition counselling"[Title/Abstract] OR "dietary feedback"[Title/Abstract] OR "hydration education"[Title/Abstract] OR "water intake"[Title/Abstract] OR "fluid intake"[Title/Abstract]) AND (child*[Title/Abstract] OR adolescent*[Title/Abstract] OR youth[Title/Abstract] OR "young athlete*"[Title/Abstract] OR "high school"[Title/Abstract] OR academy[Title/Abstract] OR U14[Title/Abstract] OR U15[Title/Abstract] OR U17[Title/Abstract] OR U19[Title/Abstract]) AND ("team sport*"[Title/Abstract] OR soccer[Title/Abstract] OR football[Title/Abstract] OR basketball[Title/Abstract] OR volleyball[Title/Abstract] OR handball[Title/Abstract] OR rugby[Title/Abstract] OR hockey[Title/Abstract] OR baseball[Title/Abstract] OR cricket[Title/Abstract] OR "American football"[Title/Abstract]) AND ("nutrition knowledge"[Title/Abstract] OR "dietary intake"[Title/Abstract] OR "eating habits"[Title/Abstract] OR "diet quality"[Title/Abstract] OR "body composition"[Title/Abstract] OR hydration[Title/Abstract] OR "urine specific gravity"[Title/Abstract] OR performance[Title/Abstract]) | 56 |
| Scopus | 3 July 2026 | TITLE-ABS-KEY( ("nutrition education" OR "nutritional education" OR "sports nutrition education" OR "dietary intervention" OR "nutrition intervention" OR "nutrition counselling" OR "dietary feedback" OR "hydration education" OR "water intake" OR "fluid intake") AND (child* OR adolescent* OR youth OR "young athlete*" OR "high school" OR academy OR U14 OR U15 OR U17 OR U19) AND ("team sport*" OR soccer OR football OR basketball OR volleyball OR handball OR rugby OR hockey OR baseball OR cricket OR "American football") AND ("nutrition knowledge" OR "dietary intake" OR "eating habits" OR "diet quality" OR "body composition" OR hydration OR "urine specific gravity" OR performance) ) | 151 |
| Web of Science Core Collection | 3 July 2026 | TS=("nutrition education" OR "nutritional education" OR "sports nutrition education" OR "dietary intervention" OR "nutrition intervention" OR "nutrition counselling" OR "dietary feedback" OR "hydration education" OR "water intake" OR "fluid intake") AND TS=(child* OR adolescent* OR youth OR "young athlete*" OR "high school" OR academy OR U14 OR U15 OR U17 OR U19) AND TS=("team sport*" OR soccer OR football OR basketball OR volleyball OR handball OR rugby OR hockey OR baseball OR cricket OR "American football") AND TS=("nutrition knowledge" OR "dietary intake" OR "eating habits" OR "diet quality" OR "body composition" OR hydration OR "urine specific gravity" OR performance) | 87 |
| SPORTDiscus/EBSCO | 3 July 2026 | XB ("nutrition education" OR "nutritional education" OR "sports nutrition education" OR "dietary intervention" OR "nutrition intervention" OR "nutrition counselling" OR "dietary feedback" OR "hydration education" OR "water intake" OR "fluid intake") AND XB (child* OR adolescent* OR youth OR "young athlete*" OR "high school" OR academy OR U14 OR U15 OR U17 OR U19) AND XB ("team sport*" OR soccer OR football OR basketball OR volleyball OR handball OR rugby OR hockey OR baseball OR cricket OR "American football") AND XB ("nutrition knowledge" OR "dietary intake" OR "eating habits" OR "diet quality" OR "body composition" OR hydration OR "urine specific gravity" OR performance) | 41 |
| Cochrane CENTRAL | 3 July 2026 | ("nutrition education" OR "nutritional education" OR "sports nutrition education" OR "dietary intervention" OR "nutrition intervention" OR "nutrition counselling" OR "dietary feedback" OR "hydration education" OR "water intake" OR "fluid intake"):ti,ab,kw AND (child* OR adolescent* OR youth OR "young athlete*" OR "high school" OR academy OR U14 OR U15 OR U17 OR U19):ti,ab,kw AND ("team sport*" OR soccer OR football OR basketball OR volleyball OR handball OR rugby OR hockey OR baseball OR cricket OR "American football"):ti,ab,kw AND ("nutrition knowledge" OR "dietary intake" OR "eating habits" OR "diet quality" OR "body composition" OR hydration OR "urine specific gravity" OR performance):ti,ab,kw | 35 |
| Total | — | — | 370 |
