# Supplementary material for: Nutrition education and behavioral interventions in youth team-sport and related development settings: a systematic review of nutrition knowledge, dietary behaviors, hydration, body composition, and performance
Source: Front Nutr. 2026 Aug 24;13:1939289. doi: 10.3389/fnut.2026.1939289 (PMC13547255; doi:10.3389/fnut.2026.1939289)
Supplement: Supplementary file 2 [file Table_2.docx]

**Table S2.** Excluded and non-retrieved reports with reasons.

| Excluded report | Exclusion category | Reason for exclusion |
| --- | --- | --- |
| Chapman P, Toma RB, Tuveson RV, Jacob M. Nutrition knowledge among adolescent high school female athletes. Adolescence. 1997;32(126):437-446. PMID: 9179339. | Full text unavailable | Potentially eligible based on title and abstract because it evaluated a sport nutrition education programme in adolescent female athletes, but the full text could not be retrieved for eligibility confirmation and data extraction. |
| Palmer MS, Logan HM, Spriet LL. On-ice sweat rate, voluntary fluid intake, and sodium balance during practice in male junior ice hockey players drinking water or a carbohydrate-electrolyte solution. Appl Physiol Nutr Metab. 2010;35(3):328-335. doi:10.1139/H10-027. | Acute protocol not aligned with review scope | The study evaluated sweat rate, voluntary fluid intake, sodium balance, and a carbohydrate-electrolyte solution during hockey practice. It did not assess a nutrition education, counselling, or behaviour-change intervention. |
| Carvalho P, Oliveira B, Barros R, Padrão P, Moreira P, Teixeira VH. Impact of fluid restriction and ad libitum water intake or an 8% carbohydrate-electrolyte beverage on skill performance of elite adolescent basketball players. Int J Sport Nutr Exerc Metab. 2011;21(3):214-221. doi:10.1123/ijsnem.21.3.214. | Acute protocol not aligned with review scope | The study compared fluid restriction, water intake, and a carbohydrate-electrolyte beverage during basketball sessions. Although hydration knowledge/behaviour was measured, the intervention was an acute intake condition rather than an educational or behavioural programme. |
| Dougherty KA, Baker LB, Chow M, Kenney WL. Two percent dehydration impairs and six percent carbohydrate drink improves boys basketball skills. Med Sci Sports Exerc. 2006;38(9):1650-1658. doi:10.1249/01.mss.0000227640.60736.8e. | Acute protocol not aligned with review scope | The study focused on dehydration and carbohydrate-electrolyte beverage effects during basketball performance testing. It was excluded because it did not evaluate a structured nutrition education or behaviour-change intervention. |
| Stover EA, Zachwieja J, Stofan J, Murray R, Horswill CA. Consistently high urine specific gravity in adolescent American football players and the impact of an acute drinking strategy. Int J Sports Med. 2006;27(4):330-335. doi:10.1055/s-2005-865667. | Acute protocol not aligned with review scope | The study evaluated hydration status and an acute drinking strategy in American football players, but did not include an eligible nutrition education, counselling, or behavioural intervention component. |
| Baker LB, Dougherty KA, Chow M, Kenney WL. Progressive dehydration causes a progressive decline in basketball skill performance. Med Sci Sports Exerc. 2007;39(7):1114-1123. doi:10.1249/mss.0b013e3180574b02. | Acute protocol not aligned with review scope | The study assessed progressive dehydration and basketball skill performance under controlled hydration conditions. It was excluded because it was an acute experimental protocol, not a nutrition education or behavioural intervention. |
| Baker LB, Heaton LE, Nuccio RP, Stein KW. Dietitian-observed macronutrient intakes of young skill and team-sport athletes: adequacy of pre, during, and postexercise nutrition. Int J Sport Nutr Exerc Metab. 2014;24(2):166-176. doi:10.1123/ijsnem.2013-0132. | No eligible intervention | The study was an observational assessment of macronutrient intake around exercise in young skill/team-sport athletes. It did not test an intervention. |
| Pai NN, Brown RC, Black KE. Risk of low energy availability and nutrition knowledge among female team sport athletes. J Sports Med Phys Fitness. 2024;64(5):446-454. doi:10.23736/S0022-4707.23.15273-X. | No eligible intervention | The study used a cross-sectional design to examine low energy availability risk and nutrition knowledge in female team-sport athletes. It did not evaluate an intervention. |
| Poveda Loor CL, Morán Zuloaga JL, Vélez Zuloaga NX, Guerrero Haro JJ, Guijarro Jara AF, Rocha Vélez KS, Raffo Domínguez JG, Sierra Nieto VH. Perfil antropométrico, somatotipo y nivel de conocimiento en nutrición en futbolistas de categorías formativas. Retos. 2026;75:420-433. doi:10.47197/retos.v75.118150. | No eligible intervention | The study assessed anthropometry, somatotype, and nutrition knowledge in youth soccer players using a descriptive/analytical design. It did not include an intervention. |
| McDonald CV, McSorley EM, McNeilly AM, Magee PJ. Nutritional knowledge of team sport athletes. J Nutr Sci. 2026;15:e39. doi:10.1017/jns.2026.10107. | No eligible intervention | The study assessed nutrition knowledge in team-sport athletes but did not evaluate the effect of a nutrition education, counselling, or behaviour-change programme. |
| Tomljanovic M, Kezic A, Tomljanovic M, Čačić Kenjerić D. Sports nutrition knowledge and carbohydrate intake in young male elite football players: insights from a case study of HNK Hajduk Academy. J Funct Morphol Kinesiol. 2025;10(2):169. doi:10.3390/jfmk10020169. | No eligible intervention | The study assessed sports nutrition knowledge, Mediterranean diet adherence, carbohydrate intake, and match-related performance indicators in academy football players. It did not include an intervention. |
| Cole M, Carter JL, Brooks F, Roberts C. Caregivers nutrition knowledge and perspectives on the enablers and barriers to nutrition provision for male academy football players. J Int Soc Sports Nutr. 2025;22(1):2495879. doi:10.1080/15502783.2025.2495879. | Ineligible population or target | The study assessed nutrition knowledge and barriers/enablers among parents and host families of academy football players. It was excluded because the target population was caregivers rather than young athletes receiving an intervention. |
| Martínez-Rodríguez A, Vicente-Martínez M, Sánchez-Sánchez J, Miralles-Amorós L, Martínez-Olcina M, Sánchez-Sáez JA. Eating disorders in top elite beach handball players: cross sectional study. Children (Basel). 2021;8(3):245. doi:10.3390/children8030245. | No eligible intervention | The study was cross-sectional and focused on eating disorder risk and body composition in beach handball players. It did not test a nutrition intervention. |
| Kaplan A, Ceylan B, Baydil B, Simenko J. Hydration and fluid intake in basketball players during training: comparison of different age categories. Appl Sci. 2025;15(19):10304. doi:10.3390/app151910304. | No eligible intervention | The study compared hydration status and fluid intake between basketball age categories during training. It was excluded because it was observational and did not include an intervention. |
| Uylas E, Mancı E, Rodriguez-Sanchez N, Bediz CŞ, Günay E. The effect of fluid restriction and intake conditions on the shooting performance of competitive adolescent handball players. Nutrients. 2024;16(23):4246. doi:10.3390/nu16234246. | Acute protocol not aligned with review scope | The study examined the effects of fluid restriction and fluid intake conditions on shooting performance in adolescent handball players. It was excluded because the intervention was an acute hydration condition, not a nutrition education or behavioural programme. |
| Timnea-Florescu AC, Dinulescu A, Prejmereanu A, Timnea OC, Nemes AF, Nemes RM. The effect of dietary supplementation on physical performance in adolescent male soccer players infected with SARS-CoV-2. Nutrients. 2025;17(3):527. doi:10.3390/nu17030527. | Ineligible population or target | The study evaluated dietary supplementation in adolescent soccer players infected with SARS-CoV-2. It was excluded because the intervention was linked to post-infection recovery rather than the general nutrition education/behaviour-change scope of this review. |
| Layouni S, Ksibi S, Ach T, Elmtaoua S, Ceylan Hİ, Ghali H, Tiss B, Ajili MA, Jemni S, Muntean RI, Dergaa I. A cross-sectional assessment of nutritional knowledge gaps and feasibility of digital intervention among adolescents soccer players in Tunisian elite club. Nutrients. 2025;17(22):3598. doi:10.3390/nu17223598. | Ineligible publication type or insufficient intervention report | The study assessed nutritional knowledge gaps and feasibility of a digital intervention among adolescent soccer players, but did not evaluate the effect of a completed nutrition education intervention on eligible outcomes. |
| Wade J, Sotos-Prieto M, Orben K, Gonzalez-Rodenas J, Brannan R. Sports nutrition knowledge in adolescent athletes. J Acad Nutr Diet. 2019;119(9 Suppl):A46. doi:10.1016/j.jand.2019.06.125. | Ineligible publication type or insufficient intervention report | Conference abstract reporting a nutrition education intervention in youth academy soccer players. It was excluded because it did not provide a full peer-reviewed intervention report with sufficient methodological and outcome detail for extraction. |
| Griffin J. Nutrition for the serious young athlete -- the educational approach. SportEX Dynamics. 2007;(12):7-9. | Ineligible publication type or insufficient intervention report | Educational/commentary article describing a nutritional education approach for young athletes, without an empirical intervention study or extractable pre-post outcome data. |
| Martín-Payo R, Fernández-Álvarez M del M, Zabaleta-del-Olmo E, García-García R, González-Méndez X, Carrasco-Santos S. Feasibility Study of an Educational Intervention to Improve Water Intake in Adolescent Soccer Players: A Two-Arm, Non-Randomized Controlled Cluster Trial. Int J Environ Res Public Health. 2021;18(3):1339. doi:10.3390/ijerph18031339. | Overlapping/duplicate report | Secondary report from the same underlying cohort as Fernández-Álvarez et al. (2020): Asturias, adolescents aged 13–16 years, five team/club clusters, 2018–2019 season, and a six-month Behaviour Change Wheel intervention using a web-app and posters. It was linked to the primary report and excluded from the unique-study count to prevent double counting. |
